# Revisiting the clinico-radiological features of an unusual inguino-labial swelling in an adult female

**DOI:** 10.1016/j.ijscr.2022.107515

**Published:** 2022-08-17

**Authors:** T. Vinoth, Ankit Lalchandani, Swastik Bharadwaj, Bharati Pandya

**Affiliations:** Department of General Surgery, All India Institute of Medical Sciences, Bhopal, Madhya Pradesh, India

**Keywords:** Hydrocele of canal of Nuck, Patent processus vaginalis, Fluctuation, Transillumination, Inguinolabial swelling

## Abstract

**Introduction:**

Processus vaginalis in females or the canal of Nuck was first described by the Dutch anatomist Anton Nuck in 1691 (Ranschaert and Worsley, n.d.; Brainwood et al., 2020 [1,2]). Its patency after birth predisposes to congenital presentations like hydroceles, encysted hydroceles and hernias presenting as an unusual inguino-labial swelling. It gets obliterated by first year of life and hence anomalies related to its patency in adult hood are even rare. It is hence important to be familiar with the clinico-radiological aspects of such a presentation when it is encountered.

**Case report:**

Case presentation is of an adult woman of 36 years with hydrocele of the canal of Nuck.

**Discussion:**

We proceed to discuss the clinico-radiological features and variations of this entity in our case report.

**Conclusion:**

In conclusion, differential diagnosis of female inguino-labial swellings need detailed evaluation to exclude the possibility of rare entities.

## Highlights

• Adult Hydrocele of Canal of Nuck is an uncommon entity.
• It poses diagnostic dilemma in many circumstances.
• An appropriate assessment is the key to diagnosis.

## 1 Introduction

A variety of differential diagnoses form a part of inguino-labial swellings and are at times easy to approach on mere clinical grounds. Elaborate investigations become mandatory in rare incidences. Hydrocele of the canal of Nuck is one such clinical entity, though first described in 1691. Much required is the precision in treatment modalities and these are aided by a thorough clinico-radiological knowledge which the authors wish to emphasize and hence the case reporting.

## 2 Case report

**Fig. 1:**
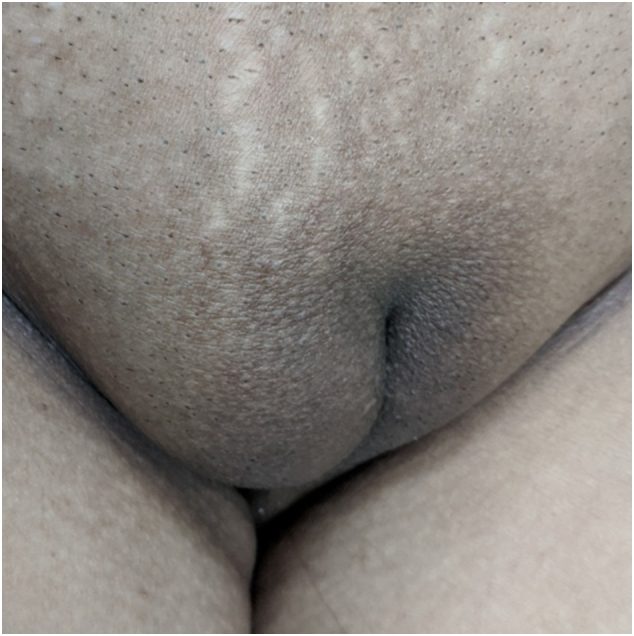
Right inguino-labial swelling.

**Fig. 2:**
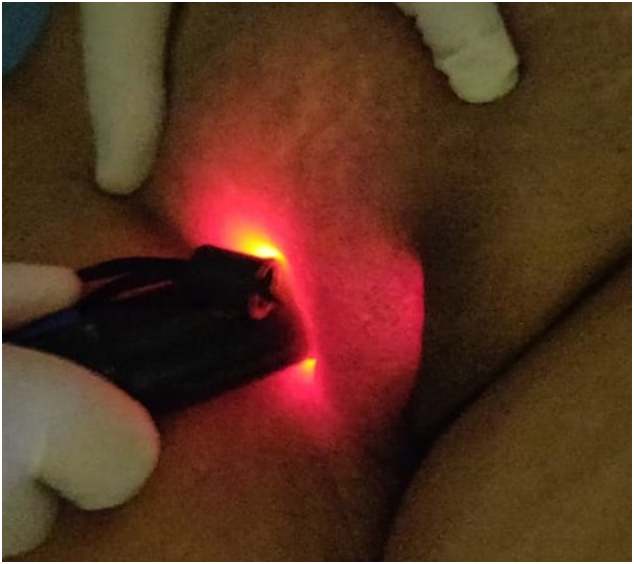
Demonstration of positive transillumination test.

A 36-year-old, married, multiparous house-wife, presented to our out patient's department with complaints of a painless swelling over right groin of 3 months duration. On examination a soft-cystic, fluctuant, right inguino-labial swelling was noted, which was transilluminant (Fig. 1, Fig. 2). There was no cough impulse and the swelling was neither reducible, nor compressible. She had no addictions to smoking, drinking etc. and had no relevant history of similar complaints in her family. She had no medical co-morbidities.

All along her case the SCARE 2020 criteria and checklist were followed and were adhered to [3].

**Fig. 3:**
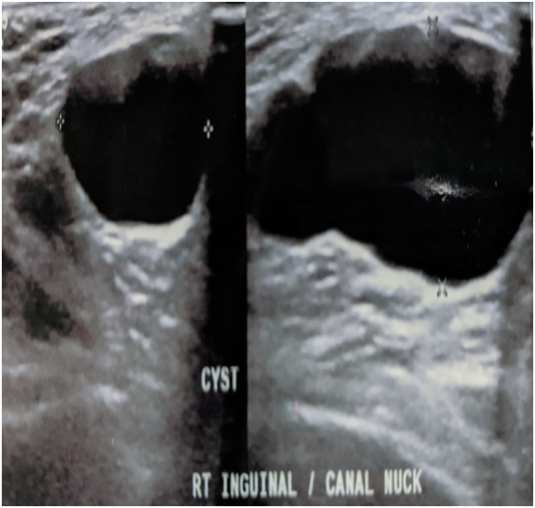
Ultrasound picture showing cyst in the canal of Nuck with septae.

**Fig. 4:**
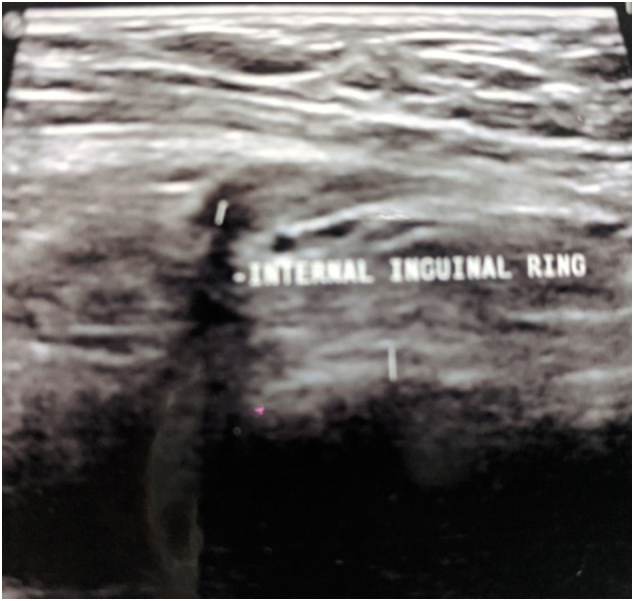
Also having a demonstrable extension of diameter 5 mm, through the deep inguinal ring.

Hematological and biochemical investigations which were performed were normal. With a clinical suspicion of Canal of Nuck cyst and differential diagnosis of a lymphatic cyst or lymphangioma, ultrasound was done. It was consistent with a cystic inguino-labial swelling, which was septated, measuring 6.3 cm × 2.2 cm × 1.9 cm (Fig. 3, Fig. 4), with a demonstrable intra-abdominal extension at the internal ring, suggestive of communicating hydrocele of the canal of Nuck. She was planned for an open surgical procedure, after detailed discussion with the patient to which she and her relations agreed, and the procedure was performed by the surgical unit chief.

**Fig. 5:**
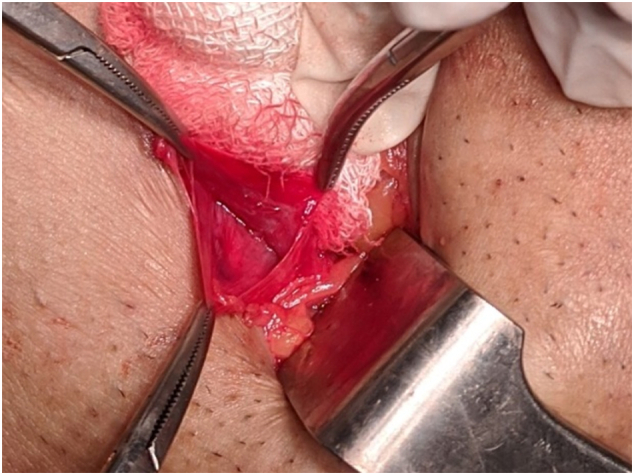
Showing intra-operative picture of proximal end communicating with the peritoneal cavity.

**Fig. 6:**
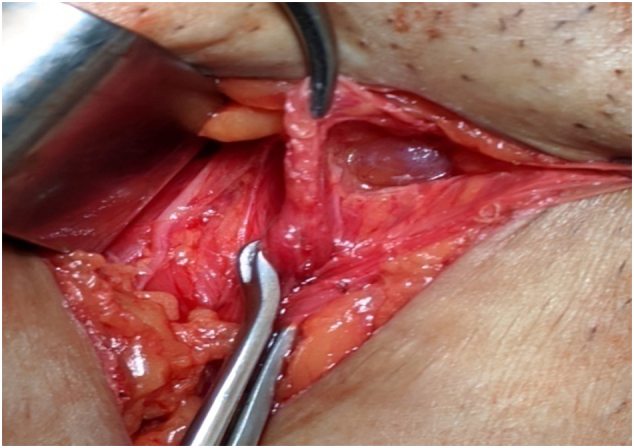
Distal ‘cyst within cyst’, septated cysts.

An inguinal incision was taken under field block and the swelling was approached like an inguinal hernial sac. The cystic sac was multi-locular and hence was dissected towards the neck first. The neck was then opened and transfixed at the internal ring and fluid as also the sac was taken for investigations. The distal part of the sac revealed cysts within cyst for which subsequent dissection was meticulously carried out to remove all the cystic spaces till the distal limit of the swelling in the labia majora. Patient tolerated the procedure well (Fig. 5, Fig. 6).

The surgery performed was herniotomy along with complete excision of the distal cystic lesion till the labia majora. Histology confirmed the sac sent to be peritoneal sac with areas of fibrosis and there was no infection nor any abnormal cells detected in the peritoneal fluid. Her post-operative period was uneventful and she was discharged the next day. One year post intervention, she continues to remain symptom-free, without any ipsilateral recurrence or contralateral swellings.

## 3 Discussion

The canal of Nuck, akin to the patent processus vaginalis in males, is an abnormal patent pouch of peritoneum, extending from anterior aspect of round ligament of uterus to the labia majora in females when it fails to obliterate completely. The quoted incidence in neonates is of 80–94 % while at one year age it drops to 30 % [1], but symptomatic cases despite the patency, are rare [2], [5]. The aim at reporting this is to highlight the clinico-radiological features.

Three types of hydrocele of canal of Nuck are encountered: i) encysted variety has no communication with the peritoneal cavity. ii) Variety with persistent communication between hydrocele and the peritoneal cavity and iii) the hour-glass variety, as a result of constriction at deep inguinal ring so that part is communicating and part is enclosed [4], [5]. If the connection with the peritoneal cavity is small, it results in a communicating hydrocele allowing only fluid to pass. If it is larger and allows abdominal contents to pass through and takes the form of a hernia [1], [2]. In between are conditions of partial obliteration resulting in encysted hydrocele of the canal of Nuck, which may or may not have a patency, but adhesions, fibrosis or the process of obliteration in them may not allow fluid to migrate back into the peritoneal cavity, resulting in either a loculated or a single cystic swelling which clinically is neither compressible nor reducible [1], [2].

The classical presentation of a painless inguinolabial swelling more commonly on the right side and occasionally bilateral, which reduces on lying down, indicates a hernia [1], [2], [4]. However hydroceles may have painful, tense and cystic nature. There are cases reported of endometriosis, ectopic pregnancy; and herniation of fallopian tubes and ovaries into the canal of Nuck and related infertility as well. Rare case of hemorrhage into the cyst has also been reported due to obstetrical trauma [5], [6], [7], [11]. Occasionally, inflammation or trauma may modify the symptomatology [1], [5], [9]. The hydroceles of communicating nature may have variation in size after lying down. But majority have obliteration at the peritoneal end and are neither compressible, nor reducible. Transillumination is seen like in our case when the fluid is clear. Fluctuation can be elicited [1], [2]. There may be rare extension of the swelling in the intraperitoneal compartment when, even cross-fluctuation may be elicited, like is seen in Hernia-en-bissac in males [7], [9].

Preoperatively, it is necessary to differentiate conditions which may create a diagnostic dilemma: femoral hernia, inguinal hernia with irreducibility, lipoma, lymphangioma, leiomyoma, sarcoma and occasionally round ligament varicosities, cysts, abscesses and lymph nodes may mandate use of imaging modalities [1], [2], [4].

The most commonly used modality is ultrasound. Hydrocele of the canal of Nuck typically presents as a cystic mass superficial and medial to the pubic bone at the level of the superficial inguinal ring. In the case of a hydrocele, ideally peritoneal communication is absent but in a case like ours where loculations and adhesions caused the tense cystic collection, in spite of an existing communication, there is also no change with Valsalva maneuver. Ultrasound is excellent at picking up the presence of bowel loops and ovaries, ectopic pregnancy, or endometrial tissue in the canal [1], [2].

When Ultrasound fails to elucidate findings, further investigations like CT scan or MRI may be required [1]. CT picks up hydroceles as homogeneous fluid-filled unilocular cysts, extending to the labia. The inguinal canal communication may or may not be identifiable. MRI in children is preferred as it has no radiation exposure and when there is a strong suspicion of solid lesion in the differential diagnosis. MR shows up as a thin walled tense cystic mass in the inguinal area. The wall of the hydrocele may show thick wall with mild contrast enhancement, if infected [1], [2].

Once diagnosed, most surgeons opt for a surgical approach which could be either open [8] or laparoscopic [9]. Surgical excision with ligating the sac at the internal ring is the preferred modality of treatment [10]. Meshplasty has been performed both open and laparoscopically [8], [9]. Very rarely in cases not having a substantial size or symptoms, a conservative approach of wait and watch may be used.

## 4 Conclusion

Hydrocele of the canal of Nuck is a rare entity especially in adulthood. We encountered one such case and decided to operate on her and present the clinico-radiological features of this rare inguinolabial swelling in females which needs to be borne in mind while considering the differential diagnoses.

## Provenance and peer-review

Not commissioned, externally peer reviewed.

## Funding

There was no funding from sponsors or other sources utilized for this manuscript.

## Ethical approval

Written informed consent was obtained from the patient for publication of this case report and accompanying images. A copy of the written consent is available for review by the Editor-in-chief of this journal on request.

## Consent

Patient was taken into confidence at every step and willingly consented to get her clinical pictures published, and her identity details were not disclosed, maintaining the secrecy of her identity.

## Author contribution

The first author has actively participated in literature search and manuscript writing.

The second and third authors were involved in taking case history, admitting and working up the patient for surgery. They were assisting surgeons and were also involved in post operative care of the patient.

The fourth, corresponding author was the chief operating surgeon for this patient and envisaged the manuscript writing and supervised the entire process.

## Registration of research studies

N/A.

## Guarantor

Such rights in our publication lie with the Departmental Head, who has been kept in knowledge of our work and has approved of it.

## Declaration of competing interest

The authors declare no conflict of interest in preparing this article.

The SCARE list has been adhered to in the preparation of this manuscript.
